# Effect of Methylsulfonylmethane Pretreatment on Aceta-minophen Induced Hepatotoxicity in Rats 

**Published:** 2013-08

**Authors:** Shahab Bohlooli, Sadollah Mohammadi, Keyvan Amirshahrokhi, Hafez Mirzanejad-asl, Mohammad Yosefi, Amir Mohammadi-Nei, Mir Mehdi Chinifroush

**Affiliations:** 1 Department of Pharmacology, School of Medicine, Ardabil University of Medical Sciences, Ardabil, Iran; 2 Department of Pathology, School of Medicine, Ardabil University of Medical Sciences, Ardabil, Iran

**Keywords:** Acetaminophen, Anti-oxidant, Hepatotoxicity, Methylsulfonylmethane Poisoning

## Abstract

***Objective(s):*** Methylsulfonylmethane (MSM) is a sulfur-containing compound found in a wide range of human foods including fruits, vegetables, grains and beverages. In this study the effect of MSM pretreatment on acetaminophen induced liver damage was investigated.

***Materials and Methods:*** Male Sprague Dawley rats were pretreated with 100 mg/kg MSM for one week. On day seven rats were received acetaminophen (850 mg/kg, intraperitoneal). Twenty-four hours later, blood samples were taken to determine serum aspartate aminotransferase (AST) and alanine aminotransferase (ALT). Tissue samples of liver were also taken for the determination of the levels of malondialdehyde (MDA); total glutathione (GSH), superoxide dismutase (SOD), and myeloperoxidase (MPO) activity together with histopathological observations.

***Results:*** High dose of acetaminophen administration caused a significant decrease in the GSH level of the liver tissue, which was accompanied with a decrease in SOD activity and increases in tissue MDA level and MPO activity. Serum ALT, AST levels were also found elevated in the acetaminophen-treated group. Pretreatment with MSM for one week was significantly attenuated all of these biochemical indices.

***Conclusion:*** Our findings suggest that MSM pretreatment could alleviate hepatic injury induced by acetaminophen intoxication, may be through its sulfur donating and antioxidant effects.

## Introduction

Methylsulfonylmethane (MSM) is a sulfur-containing compound found in a wide range of human foods including fruits, vegetables, grains, and beverages (1). Recently, the anti-inflammatory effect of MSM on lipopolysaccharide-induced inflammatory responses have been shown in murine macrophages (2) and on experimental colitis in rats (3). Nevertheless, the effects of anti-oxidant of MSM on pitting edema (4) and exercise induced oxidative stress have been reported (5). It was reported that MSM may be used as a precursor for the synthesis of methionine and cysteine, sulfur containing amino acids, and act as a source of sulfur (6). 

In previous studies it has been shown that MSM is relatively non-toxic compound (7). In addition, some investigations have reported that MSM did not cause any adverse side effects or increased mortality (8, 9). Kim et al have also depicted that MSM may cause side effects not more than the placebo (10).

Acetaminophen (APAP) is a safe, effective and widely used analgesic–antipyretic drug. However, an overdose can induce severe hepatotoxicity (11). Recent evidences suggest that reactive metabolite formation and glutathione depletion is some of the initiating events for its toxicity (12). 

Considering the underlying mechanisms of acetaminophen induced liver injury and sulfur donating; free radical lowering effect of MSM, it seems that premedication with MSM may prevent hepatotoxicity associated with acetaminophen overdose. 

Our previous studies showed that single and acute administration of MSM does not exert a significant increase on plasma GSH level (13), however; in chronic and pretreatment mode it is able to produce significant increase in plasma GSH level (14). Therefore, the present work was undertaken as a pilot study to examine the potential protective effect of MSM pretreatment against acetaminophen induced hepatotoxicity in rats. 

## Materials and Methods

*Chemicals*

Methylsulfonylmethane and Acetaminophen was used in this study (Sigma-Aldrich Chemicals). All other chemicals were of analytical grade.

*Experimental animal and design*

Twenty four pathogen-free male Sprague Dawley rats (four weeks) were obtained f (Animal Center, Pasture Institute, Iran). Rats were then housed in specific standard laboratory conditions for one week. All animals were kept in a temperature-controlled environment (25 ± 1 °C), a relative humidity (70 ± 5%), and were fed with standard rat chow diet and water *ad libitum*. Rats weighting in the range of 190–220 g were used for induction of APAP-induced hepatotoxicity. All rats were received humane care in accordance to the “Guide for the Care and Use of Laboratory Animals” (National Academies Press, Washington, DC, USA, 1996).

The experiment was conducted according to the procedures described previously (15). Acetaminophen was dissolved in 40% polyethylene glycol 400 for intraperitoneal (IP) administration. For all groups, the time interval between first and second administration at day seven was 30 min. Rats were randomly divided into four groups, each consisting of six rats. Group 1 served as normal control and received 1 ml/kg isotonic 0.9% NaCl IP daily for seven days, and then injected IP with 10 ml/kg isotonic 0.9% NaCl, at day seven. Group 2 served as hepatotoxicity control and received 1ml/kg isotonic 0.9% NaCl IP daily for seven days, and at the day seven were intoxicated with 835 mg/kg acetaminophen IP. Group 3 used as MSM control and received 100 mg/kg MSM IP in water daily for seven days and then injected with 10 ml/kg isotonic 0.9% NaCl IP. Group 4 was received 100 mg/kg MSM IP in water daily for seven days and at day seven intoxicated with 835 mg/kg acetaminophen IP. After 24 hr of acetaminophen intoxication, rats were euthanized by ether and then sacrificed. Blood sample was collected by cardiac puncture in heparinized tubes. The liver was immediately taken out and washed with ice-cold saline, then weighed and stored at −80 °C. The blood and liver samples were then assessed for their biochemical indices. A piece of liver was finally fixed in formalin to examine histopathological changes. 

*Measurement of liver function markers*

The whole blood was centrifuged at 3000 rpm for 10 min to separate the plasma. Markers of liver function including AST and ALT were measured with colorimetric methods using commercially available kits (Zist-Shimi Co, Tehran, Iran) by double beam spectrophotometer (T80+, PG-Instruments, UK).

*Measurement of antioxidant enzymes and lipid peroxidation*

Liver tissues were homogenized in four volumes of ice-cold 150 mM Tris–HCl (pH 7.4) using Hiedolph homogenizer (SilentCrush M, Hiedolph, Germany). The homogenates were centrifuged at 16000 g for 15 min at 4C to obtain a supernatant for various biochemical analyses. Lipid peroxidation in the liver homogenate was determined by the formation of MDA and was measured as reported previously (14). The data are expressed in nanomolar (nmol) of MDA per milligram of liver tissue (nmol/mg tissue). Liver Total GSH and SOD were determined as markers of anti-oxidant capacity using commercially available kits (Cayman, USA) and described in concentrations were expressed as nmol of total GSH per mg tissue. SOD activity was stated as units per mg tissue. MPO activity was then measured using commercially available kits (Cayman, USA) as index of neutrophil infiltration to liver tissue and considered as marker of inflammation and oxidative stress and expressed as units per mg tissue.

*Histopathological observation*

The liver tissues were fixed in 10% formalin buffer solution for 24 hr embedded in paraffin. The serial sections were cut 5 m thick and stained with haematoxylin-eosin (HE), and then observed for the changes of liver injury by photomicroscope.

*Statistical analysis*

All data were expressed as means ± standard deviations. Statistical analysis was performed using one-way ANOVA followed by Tukey test. *P*< 0.05 was considered significant. 

## Results

**Figure 1:**
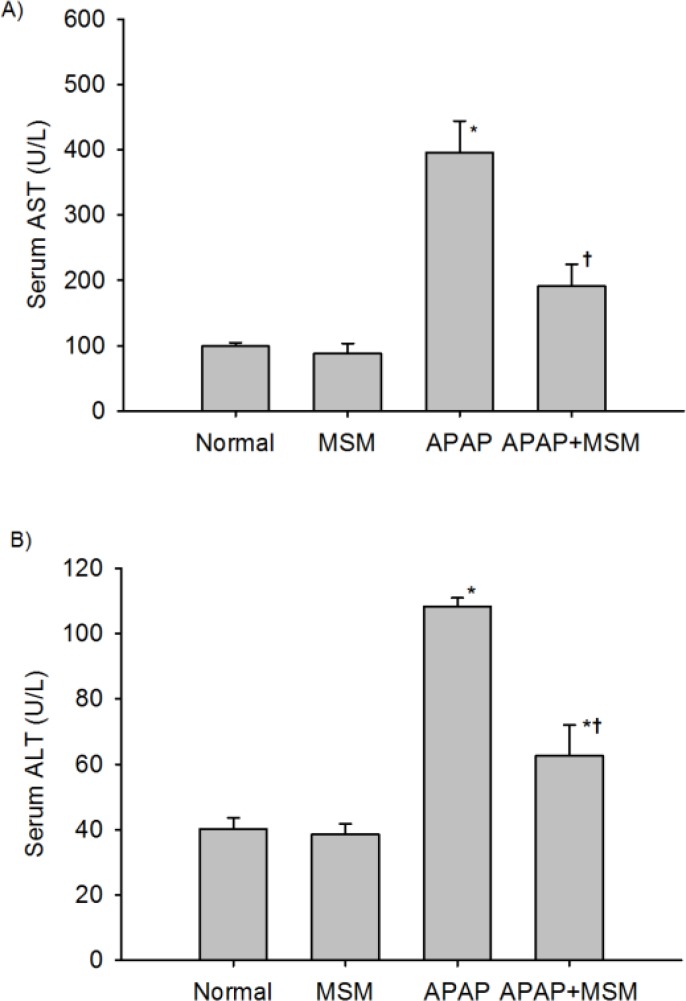
Effect of pretreatment with MSM 100 mg/kg on serum A) AST and B) ALT in rats with acetaminophen induced hepatotoxicity. Values are mean±SEM (n=6). APAP: acetaminophen; MSM: Methylsulfonylmethane

There is an increase in the levels of AST and ALT in the blood, which reflects the failure of liver function due to APAP-induced hepatotoxicity. In Figure 1, AST and ALT activities were significantly increased after the administration of APAP as compared with the normal group (*P*< 0.001). Although pretreatment with 100 mg/kg of MSM for one week significantly blunted the elevation of AST and ALT (*P*< 0.001), the serum value of ALT was still significantly higher than the normal group.

It was found that liver MDA level was significantly higher in APAP intoxicated group comparing with normal group (*P*< 0.05). Pretreatment with MSM was able to prevent MDA elevation (*P*< 0.01).

Compared with the normal group, hepatic MPO activity was increased significantly in the group who received acetaminophen (*P*< 0.01), indicating the increase in neutrophil infiltration to the tissue (Table 1). This elevation in the myeloperoxidase activity which induced by acetaminophen was significantly attenuated with MSM pretreatment (*P* < 0.05).

**Table 1:** Effect of pretreatment with MSM on liver function markers in rats with acetaminophen-induced hepatotoxicity

Treatment	MDA (nmol/mg tissue)	Total GSH (nmol/mg tissue)	SOD (U/mg tissue)	MPO (U/mg tissue)
Normal	5.01±0.59^++^	33.55±4.88^++^	1.4±0.1^+^	2.1±0.5^+^
APAP	8.29±2.26^*^	10.58±0.89^*^	0.8±0.2^*^	4.3±0.9^*^
MSM 100 mg	3.64±0.69^++^	36.03±6.19^++^	1.5±0.4^+^	2.8±1.0^+^
MSM 100 mg + APAP	4.54±1.67^++^	20.94±1.82^++*^	1.6±0.5^+^	2.6±0.5^+^

MSM: Methylsulfonylmethane; MDA: Malondialdehyde; GSH: Total glutathione; SOD: Superoxide dismutase; MPO: Myeloperoxidase

Values were expressed as mean ± SD, n=6. APAP: acetaminophen* *P* < 0.05 significantly different from normal group++ *P*< 0.01 significantly different from APAP control group+ *P*< 0.05 significantly different from APAP control group

* *P*< 0.01 versus normal group. † *P* < 0.01 versus APAP group

The endogenous antioxidant, GSH level in the hepatic tissue was decreased significantly after acetaminophen intoxication as compared to the levels measured in the normal group (*P*< 0.001) (Table 1). However, MSM pretreatment significantly reversed the acetaminophen-induced GSH reduction (*P*< 0.001), but the hepatic GSH level was still low compared to the normal group.

Activity of hepatic SOD was presented in Table 1. Compared with the normal group, hepatic SOD activity was decreased significantly in the acetaminophen group (*P*< 0.01), indicating diminished enzymatic antioxidant activity in the tissue. Pretreatment with MSM was successful to maintain the SOD activity in the hepatic tissue (*P* < 0.05) close to the range of normal group.

**Figure 2:**
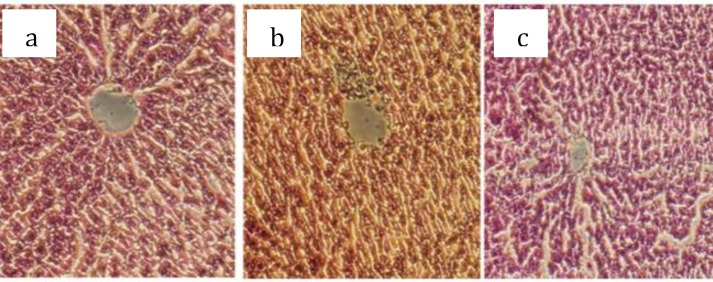
Representative photographs of liver histopathology (H & E, 200 ) in a) normal control rat, no necrosis b) acetaminophen intoxicated control rat and c) rat pretreated by MSM (100 mg/kg).

Histopathological examination of liver sections of control group showed normal cellular architecture with distinct hepatic cells, sinusoidal spaces and central vein (Figure 2a). Disarrangement of normal hepatic cells with necrosis and destruction of central vein are observed in acetaminophen-intoxicated liver (Figure 2b). The liver sections of the rat pretreated in the test group (Figure 2c), showed less necrosis and overall less visible changes compared to acetaminophen control group, showing that pre-supplementing with MSM has some protective effect on liver tissue.

## Discussion

In the present study, acetaminophen-induced liver toxicity was evidenced by biochemical measurements and histopathological observations. Increased level of serum AST and ALT indicated deterioration in the hepatic functions due to toxic effects of APAP. Pretreatment with 100 mg/kg MSM for one week helped in attenuating the acetaminophen induced toxic consequences in the liver. 

The ability of MSM to mitigate APAP induced hepatic toxicity is partly due to its serving action as a sulfur donating agent for synthesis of new cysteine, a rate limiting precursor of GSH production (6, 16). 

Acetaminophen is a safe and effective analgesic when used in therapeutic doses. However, an overdose can induce severe hepatotoxicity (17). In overdose, acetaminophen is metabolized to NAPQI (N-acetyl-p-benzoquinoneimine) predominantly via hepatic cytochromes. The NAPQI-induced depletion of cytosolic and mitochondrial GSH leads to liver injury (18) which was confirmed in our study by concurrent increase in liver MDA indicating significant lipid peroxidation. The APAP-induced decrease in GSH pool was alleviated significantly by MSM pretreatment, which is in agreement with the fact that exogenous administration of antioxidants may influence the GSH metabolism (18). Therefore, MSM may play an important role in protection against acetaminophen intoxication by modulating the cellular GSH pool. It was proposed that MSM could acts as a source of sulfur which may induce new GSH synthesis (6). The ability of MSM to prevent GSH depletion in other oxidative conditions such as exercise (5, 16) or chemically induced oxidative stress (3) has been previously reported.

It has been proposed that the acetaminophen metabolism triggers lipid peroxidation which may be responsible for liver injury (19). In the present study, a significant increase in MDA content, an index of lipid peroxidation, was observed. This increase is in parallel with reduction in liver GSH and SOD levels. Our data also showed that pretreatment with MSM significantly inhibits MDA production and decreases its level toward normal, implying decreases in lipid peroxidation and liver injury. In our previous studies, MSM showed anti-oxidant effect on acetic acid induced colitis in rats (3) and exercise induced oxidative stress in humans (5). In accordance, findings from the current study also showed that pretreatment with MSM may reduce acetaminophen induced liver damage by attenuating lipid peroxidation and glutathione depletion confirmed by histopathological observations of rat liver tissue. 

There are evidences that inflammatory cells such as neutrophils may be involved in the pathophysiology of the acetaminophen-induced liver injury (20). Myeloperoxidase is one of the most important oxygen-dependent enzymes in neutrophils which if released into local tissue or the systemic circulation, can induce oxidative stress, with variable degrees of cytotoxicity (21). It was also confirmed that estimation of tissue MPO activity is reliable indicator of inflammation (21, 22). In the current study, pretreatment with MSM was able to reduce the tissue MPO activity toward normal. Beilke et al. have reported that MSM is a strong suppressor of superoxide and hydrogen peroxide production in neutrophils (23).Therefore, It may be postulated that protective effect of MSM on liver tissue could be in part due its anti-inflammatory action which is in agreement with other studies (2, 3). 

Although the present study is the first report on MSM hepatoprotective effect, it has some limitations, which need to be addressed in ongoing investigations. First, the dose-dependent effect of MSM on acetaminophen induced hepatotoxicity in not explored in current study. Second, there is a need to address the effects of MSM on other known markers of oxidation and inflammation underlying liver damage and also there is a need to explore the mechanism that MSM does its effects.

## Conclusion

Considering the low toxicity of MSM and the findings of the current study which illustrated that MSM, as a sulfur donating and antioxidant agent, alleviates indices of hepatic injury induced by acetaminophen, it is possible to suggest that MSM as a pretreatment agent has a potential to be investigated as an agent in limiting the drug-induced oxidative damage.
